# Obesity and Bone Health: A Complex Link

**DOI:** 10.3389/fcell.2020.600181

**Published:** 2020-12-21

**Authors:** Jing Hou, Chen He, Wenzhen He, Mi Yang, Xianghang Luo, Changjun Li

**Affiliations:** ^1^Department of Endocrinology, Endocrinology Research Center, The Xiangya Hospital of Central South University, Changsha, China; ^2^National Clinical Research Center for Geriatric Disorders (Xiangya Hospital), Changsha, China; ^3^Key Laboratory of Organ Injury, Aging and Regenerative Medicine of Hunan Province, Changsha, China

**Keywords:** obesity, adipose tissue, bone health, bone remodeling, bone-related diseases

## Abstract

So far, the connections between obesity and skeleton have been extensively explored, but the results are inconsistent. Obesity is thought to affect bone health through a variety of mechanisms, including body weight, fat volume, bone formation/resorption, proinflammatory cytokines together with bone marrow microenvironment. In this review, we will mainly describe the effects of adipokines secreted by white adipose tissue on bone cells, as well as the interaction between brown adipose tissue, bone marrow adipose tissue, and bone metabolism. Meanwhile, this review also reviews the evidence for the effects of adipose tissue and its distribution on bone mass and bone-related diseases, along with the correlation between different populations with obesity and bone health. And we describe changes in bone metabolism in patients with anorexia nervosa or type 2 diabetes. In summary, all of these findings show that the response of skeleton to obesity is complex and depends on diversified factors, such as mechanical loading, obesity type, the location of adipose tissue, gender, age, bone sites, and secreted cytokines, and that these factors may exert a primary function in bone health.

## Introduction

Bone is a living tissue with metabolic functions. The size and shape of bones are accurately modeled and reshaped over a lifetime to ensure skeletal structure and integrity (Xie et al., 2014; Li et al., 2017). During bone remodeling, bone can coordinate the activities of osteoblasts, osteocytes, and osteoclasts, thus maintaining the dynamic coupling balance of bone metabolism, in which osteoblasts (bone formation) and osteoclasts (bone resorption) play pivotal roles in bone metabolism. Bone marrow mesenchymal stem cells (BMSCs) have the ability to differentiate into osteoblasts and adipocytes, which is also a dynamic balance (Li et al., 2015; Li C.J. et al., 2018). In addition, bones exhibit the characteristics of endocrine organs, which secrete a variety of hormones to participate in the endocrine cycle throughout the body. For instance, bone can secrete estrogen, androgens, follicle suppressant and other sex hormones to participate in the reproductive process, cellular senescence, and osteoporosis (OP). Moreover, bone marrow fat cells can also secrete a series of adipokine including adiponectin, leptin through autocrine, and paracrine, which play an important regulatory role in local and systemic metabolism of bone marrow. There are however many metabolic osteopathy, including OP, bone tumor, and inflammatory arthritis, where this fine equilibrium is disrupted (Sun et al., 2019).

Overweight and obesity are identified as abnormal or excessive fat accumulation that can lead to impaired health. Body mass index (BMI) is a simple ratio of height to weight that is often used to distinguish between overweight and obesity adults. The WHO defines overweight as having a BMI of 25 to <30 kg/m^2^ and obesity as ≥30 kg/m^2^. In addition, obesity can be divided into three grades, namely obese class I (30 to <35 kg/m^2^), class II (35 to <40 kg/m^2^), and class III (≥40 kg/m^2^) (Barba et al., 2004; Di Angelantonio et al., 2016). However, the diversity of Asian countries is mainly manifested in ethnic and cultural subgroups, degree of urbanization, social and economic conditions, and nutritional transformation. And compared with white people of the same age, sex, and BMI, Asians typically have a higher percentage of body fat. Therefore, the BMI cut-off points for overweight and obesity may be more applicable in Asian countries (Barba et al., 2004). Overweight and obesity are associated with risk and prognosis for certain disease states (Dalamaga and Christodoulatos, 2015).

The prevalence of obesity and obesity-related diseases is on the rise worldwide due to the socioeconomic and demographic transitions. The link between adiposity and bone health has been extensively studied, but the impact of obesity on bones has long been a subject of debate. This review will discuss the correlation between adiposity and bone-related diseases from multiple perspectives.

## Extramedullary Adipose Tissue and Bone

Adipose tissue consists mainly of fat cells that have accumulated in large numbers, which are divided into fat lobules by thin layers of loose connective tissue and widely distributed in the subcutaneous and around the internal organs. It is not only involved in body construction and energy storage, but is also a vital endocrine organ. Adipose tissue has an effect on insulin sensitivity, blood pressure level, endothelial function, fibrinolytic activity and inflammatory response, and participates in a variety of important pathophysiological processes.

Adipose tissue exerts a primary function in obesity and related diseases. According to different functions, adipose tissue can be divided into white adipose tissue (WAT), brown adipose tissue (BAT), and beige adipose tissue. WAT is an energy-storing tissue that regulates energy metabolism by secreting cytokines and hormones. Excessive accumulation of WAT in the body can cause obesity and obesity-related diseases. BAT, however, is an energy-consuming organization that is involved in non-shivering heat and diet-induced heat production. Both thermogenic mechanisms are due to the exclusive expression of uncoupled protein-1 (UCPl) in the mitochondria of brown fat cells, resulting in the uncoupling of fatty acids and ATP oxidation, ultimately causing energy dissipation in the form of heat (Del Mar Gonzalez-Barroso et al., 2001; Carobbio et al., 2017; Huang et al., 2020). Thus, BAT can be capable of fighting obesity. Beige fat is more widely distributed than the typical brown fat, which is concentrated in the interscapular storage of rodents and in the supraclavicular and mediastinal areas of humans (Frontini and Cinti, 2010; Wiedmann et al., 2017). Beige fat also possesses an innate ability to metabolize energy as heat release by non-shivering thermogenesis (Zhang et al., 2018).

### Leptin Secreted by White Adipose Tissue and Bone

White adipose tissue has a main endocrine function secreting many adipokines, especially leptin and adiponectin. These latest findings suggest that adipokines are closely related to metabolic diseases such as obesity, insulin resistance and diabetes, and play an important regulatory role in bone disease (Conde et al., 2011). Particularly, leptin and adiponectin have been shown to act directly on certain bone cells, including BMSCs, osteoblasts, and osteoclasts. Consistent evidence suggests that leptin has a direct anabolic effect on osteoblasts. Astudillo et al. (2007) observed that leptin can promote the proliferation of bone marrow stromal cells, differentiation into osteoblasts, and form mineralized nodules, but prevent the differentiation into adipocytes. Thomas (1999) have also shown that leptin leads to dose-dependent increases in mRNA and protein levels of alkaline phosphatase (ALP), osteocalcin (OC), and type I collagen. Gordeladze et al. (2002) found that continuous exposure of leptin to ilium osteoblasts contributed to collagen synthesis, cell differentiation, and *in vitro* mineralization, together with cell survival and transition to preosseous cells.

Additionally, leptin may increase local bone mass and may contribute to the link between bone formation and bone resorption. Holloway et al. (2002) found that after adding human macrophage colony stimulating factor (HM-CSF) and human soluble NF-Kappab ligand receptor activator (sRANKL) to bone culture, leptin can inhibit the formation of human peripheral blood mononuclear cells (PBMCs) and mouse spleen cells to osteoclasts. Leptin increased mRNA and protein expression of osteoprotegerin (OPG, a protein that inhibits osteoclast formation) in PBMC, suggesting that its inhibition may be realized through the RANKL/RANK/OPG pathway (Holloway et al., 2002). Leptin effectively attenuated trabecular bone loss, trabecular structure change and periosteal bone formation. Leptin also significantly reduced RANKL mRNA levels which mainly regulates osteoclast development in cultured human bone marrow stromal cells (Burguera et al., 2001; Reid et al., 2018).

In addition to having a direct anabolic effect on osteoblasts, leptin also indirectly affects bones. Numerous studies have shown that in mice lacking leptin or leptin receptors, spinal trabecular volume increases, vertebral bone mass increases, while femur bone mass decreases and femur bone marrow fat increases sharply (Ducy et al., 2000; Steppan et al., 2000; Hamrick et al., 2004). Remarkably, Steppan et al. (2000) found, compared with the control group, leptin administration significantly increased femur length, systemic bone area, bone mineral density (BMD) and bone mineral content in leptin-deficient mice (ob/ob mice). This conclusion has been confirmed in other studies as well (Hamrick et al., 2005; Bartell et al., 2011; Philbrick et al., 2017). In summary, the role of leptin in skeleton remains a highly controversial area.

### Adiponectin Secreted by White Adipose Tissue and Bone

Adiponectin, the most common adipokine in plasma, has important metabolic and anti-inflammatory effects and is rapidly becoming a valuable marker for treatment in metabolic diseases. The role of adiponectin in certain bone cells including osteoblasts and osteoclasts, has been extensively studied. Human adiponectin promoted bone formation of primary human maxillary BMSCs through the APPL1-P38 MAPK pathway (Pu et al., 2016). Also, adiponectin induced osteogenesis of MSCs through adipoR1-mediated phosphorylation of P38 MAPK. Then, p38 MAPK phosphorylated c-Jun, which enhanced COX-2 (cyclooxygenase-2) expression and ultimately lead to an increase in BMP2 expression (bone morphogenetic protein 2, a strong osteogenic cytokine) (Lee et al., 2009; Huang et al., 2010). Similarly, Luo et al. (2005) demonstrated that adiponectin promoted osteoblastic proliferation, leading to increased alkaline phosphatase (ALP) activity, generation of type I collagen and OC, and increase in mineralized substrates in a dose-dependent and time-dependent manner. The increase of ALP, type I collagen, and OC is a marker of osteoblast differentiation and maturation, while matrix mineralization is a marker of osteoblastic phenotype. This study also provided evidence that adiponectin induced the proliferation and differentiation of human osteoblasts, which was realized through AdipoR/JNK pathway, while the differentiation was realized through AdipoR/P38 pathway (Sowa et al., 2002; Nöth et al., 2003; Luo et al., 2005).

Adiponectin also directly regulates osteoclast function. Adiponectin down-regulated expression of osteoclast regulators such as acid tartrate – resistant phosphatase and cathepsin K. Adiponectin also enhanced osteoclast apoptosis and reduced osteoclast precursor cells survival/proliferation (Tu et al., 2011; Pal China et al., 2018).

Significantly, like leptin, *in vivo* studies showed that the regulation of bone metabolism by adiponectin is also contradictory. Several studies reported that compared with wild-type mice, adiponectin knockout mice showed lower bone mineralization and bone density (Shinoda et al., 2006; Naot et al., 2016). But, others have shown that lack of adiponectin exert a protective effect on skeleton. Pal China et al. (2018) found KO mice prevented trabecular bone loss in mice caused by ovariectomy, and showed better bone quality (Williams et al., 2009). Taken together, the data from *in vitro* and *in vivo* studies fail to provide any definitive conclusions about the relationship between leptin, adiponectin and bone. And, white adipocytes can not only synthesize and secrete hormones including adiponectin and leptin, but also secrete proinflammatory factors such as TNF-α, IL-6, which may negatively regulate bone metabolism, further complicating the relationship between obesity and bone (Fruhbeck et al., 2001).

### Brown Adipose Tissue and Bone

It is well known that BAT not only exists in newborns, but also exists in adults, and gradually decreases with age. In fact, Ponrartana et al. (2012) have observed a significant correlation between BAT volume and cross-sectional bone size, regardless of gender. Moreover, recent findings suggest that BAT activity is positively correlated with skeletal metabolism. In contrast, some previous studies have shown that BAT can secrete fibroblast growth factor 21 (FGF21) and increase plasma FGF21 levels. Wei et al. showed that in animal models, transgenic mice overexpressing FGF21 had a lower bone mass phenotype, while mice given the drug dose of FGF21 also had a lower bone mass, because of reduced bone formation and significantly increased bone absorption (Wei et al., 2012). Also, Fazeli et al. (2015) found that FGF21 was negatively correlated with trabecular microstructural parameters, such as trabecular number (TbN), in patients with anorexia nervosa (AN). Recently, plasma FGF21 level was found to be negatively correlated with bone density in femoral neck and Ward’s triangle of hip region (Hao et al., 2018). Taken together, these data reveal that FGF21 is a major negative regulator of bone mass.

Interestingly, as an endocrine organ, bone can also secrete a variety of bioactive substances to control energy metabolism in adipose tissue. OC, a small molecular protein secreted by osteoblasts, is a classic indicator of bone formation. Li Q. et al. (2018) verified the specific role of OC in thermogenesis of brown adipocytes. Their study showed that the OC signaling pathway directly promoted the activation of the Gprc6a Tcf7 Ucp1 promoter through the positive feedback of the interaction between the Wnt3a Tcf7 and the WNT/β-catenin pathway (Li Q. et al., 2018).

In addition, bone regulates browning and energy metabolism via the expression of peroxisome proliferator-activated receptor (PPAR) in mature osteoblasts and osteocytes. Several studies have confirmed that, in bone, PPAR plays a negative regulatory role in bone formation by inhibiting osteoblast generation and promoting osteoclast activity. Specific ablation of PPAR in mature osteoblasts and osteocyte mice (OCY-PPAR-/-) was found to induce high-bone mass and low fat mass phenotypes, as well as increase fat browning and energy comsumption. Moreover, Ocy-PPARγ-/- could partly prevent the dysmetabolism caused by high fat intake (Brun et al., 2017). Interestingly, BMP7 levels were also higher in Ocy-PPARγ-/- mice in conditioned medium and serum. BMP7, derived from osteocytes, is believed to promote browning and reduce steatosis through endocrine mechanisms (Kinoshita et al., 2007; Sugimoto et al., 2007; Tseng et al., 2008; Boon et al., 2013).

Collectively, these results indicate that bone and adipose tissue can interact to regulate bone metabolism and energy metabolism, which provides a new idea for the exploration and development of treatment methods for bone-related diseases.

## Bone Marrow Adipocytes and Bone

Bone marrow adipose tissue (BMAT), which accounts for about 8% of total fat mass, is an important fat depot in the adult body and exerts a significant function in bone homeostasis and energy metabolism throughout the body (Tencerova et al., 2019). BMAT is thought to be negatively correlated with bone density and bone integrity, so it may be an important regulator of bone turnover (Fazeli et al., 2013). Studies have shown that BMAT in the lumbar spine is an independent predictive factor of fracture (Wehrli et al., 2000). BMAT is distinct from WAT and BAT. Excessive accumulation of lipids interferes with the normal function of cells and tissues, a condition known as lipotoxicity (Carobbio et al., 2017; Piccolis et al., 2019). Lipotoxicity is caused primarily by bone marrow fat through the secretion of adipokines and free fatty acids (mainly palmitate). In bone marrow, lipotoxicity is mainly manifested by the toxic effect of palmitate on bone cells, especially osteoblasts (Al Saedi et al., 2020). Exposure to adipocyte secretory factors can reduce the ability of BMSC to differentiate into bone cells, but also promote fat formation, a phenomenon that may be explained by a variety of mechanisms, including oxidative stress and proinflammatory mediators (TNF-α and IL-6) and adipokines (Horowitz et al., 2017; Singh et al., 2018). Furthermore, Elbaz et al. (2010) observed that, by co-culturing normal human osteoblasts (NHOst) with pre-differentiation adipocytes in the absence of a fatty acids synthase inhibitor (cerulenin), the differentiation and functional levels of NHOst were significantly reduced due to the lower mineralization and decreased expression of ALP, osterix, OC, and Runx2 (Elbaz et al., 2010). Actually, some evidences, indicates that palmitate has negative effects on osteoblast differentiation, bone nodule formation and mineralization.

Apart from BMSC and osteoblasts, osteoclasts are also affected by bone marrow fat to increase bone resorption and decrease bone mass (Singh et al., 2018). Takeshita et al. (2014) found that, during the differentiation of marrow stromal cells into adipocytes, RANKL expression was induced, along with the down-regulation of osteoprotegerin. The early adipogenic transcription factors C/EBPβ and C/EBPδ could bind to the RANKL promoter and ultimately stimulate RANKL gene transcription (Takeshita et al., 2014; Paccou et al., 2015; Hardouin et al., 2016).

Overall, these findings point to metabolic differences between bone marrow fat and peripheral fat that may be related to the development of therapeutic strategies for metabolic bone disease.

## Anorexia Nervosa, Obesity, and Bone

Anorexia nervosa is a major mental disorder that mainly affects women. Patients are unable to maintain normal weight due to extreme self-imposed starvation, which is typical of chronic malnutrition (Kaye et al., 2020). Bone loss is almost the most common feature in many comorbidities associated with this disease.

Interestingly, compared with normal-weight women, anorexic patients showed less subcutaneous adipose tissue (SAT) and visceral adipose tissue (VAT), but more BMAT (Bredella et al., 2009). Previous studies have shown that increased BMAT level has clinical significance and is related to bone density and bone strength. In the above content, we mentioned that bone marrow fat can inhibit bone formation and promote bone resorption. In addition, marrow fat content increased in elderly patients with OP and was inversely correlated with bone density in healthy white women, and also in obese women (Justesen et al., 2001; Shen et al., 2007; Bredella et al., 2011b). Similarly, in ovariectomy animals, loss of bone mass was often accompanied by abnormal accumulation of bone marrow fat (Bredella et al., 2009; Li S. et al., 2018; Beekman et al., 2019). Given the negative correlation between marrow fat and BMD, BMAT may be an important risk factor for low bone density and increased fracture risk in patients with AN. For example, increased BMAT and decreased bone density and bone strength were observed in AN in women and adolescents (Singhal et al., 2018; Fazeli et al., 2019). Moreover, bone marrow fat in L4 vertebral body, femoral metaphyseal and diaphyseal increased markedly in women with AN. Importantly, marrow fat content had a highly significant negative association with BMD in multiple bone sites including the lumbar spine, hip and whole body (Bredella et al., 2009). One reason may be that osteoblasts and fat cells are derived from the mesenchymal stem cells in the bone marrow, the increased adipogenic differentiation may lead to a decreased osteogenic differentiation in BMSCs.

However, studies have shown that in patients recovering from AN, BMAT levels in L4 vertebrae are similar to those in healthy controls (Fazeli et al., 2012). BMAT positively correlated with VAT in overweight/obese women, while no such association was observed in underweight women (Bredella et al., 2011b; Polineni et al., 2020). These data may indicate that BMAT is a dynamic depot, which may play different roles under the condition of insufficient and adequate nutrition. In addition, since hypercortisolemia and hypoestrogenism are both characteristics of AN, these factors may result in marrow adiposity (Putignano et al., 2001). In summary, during chronic starvation, while other fat stores are used as energy sources, this adipose tissue storage is retained, suggesting that it may have significant functions. And, studying this paradox may lead to a better understanding of the function of bone marrow fat.

## Type 2 Diabetes, Obesity and Bone

Diabetes is a chronic disease characterized by neuropathy, nephropathy and retinopathy, with type 2 diabetes mellitus (T2DM) as the predominant type. The patient presents primarily with insulin resistance, and relative insulin deficiency (Compston, 2018; Su et al., 2019). This has been accompanied by a sharp rise in the incidence of obesity, an important determinant of type 2 diabetes. Abnormal accumulation of fat both inside and outside adipose tissue not only results in structural and functional disorders (insulin resistance) of adipose tissue itself, but also affects muscles, liver, and pancreas (Tsatsoulis et al., 2013; de Araujo et al., 2017). The bone disease of type 2 diabetes remains a mystery because the mechanism by which diabetes affects the bone is multifactorial, including factors such as obesity, hyperinsulinemia and insulin-like growth factors (IGFs). The effect of insulin on bone metabolism is controversial. Studies *in vitro* have reported that insulin signals in osteoblasts can promote bone absorption (Ferron et al., 2010). Consistent with *in vitro* results, Srikanthan et al. also confirmed that insulin resistance, especially hyperinsulinemia, may adversely affect femoral neck strength (Srikanthan et al., 2014). However, other *in vivo* studies show that insulin stimulates osteoblasts to proliferate and increases the histologic markers of bone formation (Shanbhogue et al., 2016). Furthermore, in the early stages of diabetes, obesity and hyperinsulinemia often occur together, and it is challenging to identify the unique contribution of obesity and hyperinsulinemia to bone health.

It is believed that IGFs is associated with the pathogenesis of diabetic complications. In addition, serum IGF-I level was decreased in patients with poor blood glucose control (Thrailkill, 2000; Kanazawa et al., 2012). IGF I is thought to have anabolic effects on bone. Circulating IGF-I activates bone remodeling and plays an anabolic role in bone tissue (Johansson et al., 1992; Zhang et al., 2002). Some clinical studies have suggested that low serum IGF-I level is related to a higher risk of fractures independent of BMD in patients with type 2 diabetes (Ardawi et al., 2013; Miyake et al., 2017). Serum IGF-I level was found to be significantly negatively correlated with the prevalence of vertebral fractures in postmenopausal women with type 2 diabetes (Kanazawa et al., 2011, 2018). Based on the above evidence, the reduced serum IGF-I may be involved in diabetes-induced bone fragility and can be used to assess the risk of fracture in patients with T2DM.

In addition to the factors described above, the effects of hypoglycemic drugs on bone metabolism should also be taken into account. Most studies have shown that sulfonylureas and metformin have protective or neutral effects on fracture risk (Vestergaard et al., 2005; Kahn et al., 2006; Monami et al., 2008; Borges et al., 2011). In contrast, thiazolidinediones activate PPARγ, which in turn stimulate adipogenesis and inhibit osteogenesis (Gimble et al., 1996; Lecka-Czernik, 2017). Studies have reported an increased risk of fracture in patients taking thiazolidinedione (Dormuth et al., 2009; Zhu et al., 2014). Incretin hormones are considered to have the potential to treat type 2 diabetes. For instance, glucagon-like peptide-1 (GLP-1) targets primarily pancreatic β cells, where it stimulates insulin production, which helps control blood glucose levels (Baggio and Drucker, 2007). More recently, there has been growing evidence that incretins may also be beneficial for skeletal strength. GLP-1 plays a key role in bone homeostasis, inhibiting bone resorption and stimulating bone formation in response to nutrient intake (Shanbhogue et al., 2016). Indeed, GLP-1 receptor knockdown in mice can result in dramatic changes in trabecular and cortical microstructures, as well as adverse effects on bone tissue material properties (Mabilleau et al., 2013; Mansur et al., 2015, 2016). Thus, the effect on bone health should be considered when selecting hypoglycemic drugs to avoid inducing or exacerbating fractures and bone diseases in patients.

## Obesity and Bone-Related Diseases

### Osteoporosis

Osteoporosis is a skeletal metabolic disorder with multiple causes, characterized by bone loss, microstructure degeneration, increased brittleness, reduced bone strength, and increased risk of fracture. Therefore, OP seriously affects the quality of life and living standards of patients (Khaliq et al., 2016; Pagnotti et al., 2019; Yang et al., 2019).

Bone mineral density is a commonly used indicator in the diagnosis of OP, which is performed by dual energy X-ray bone absorptiometry (DEXA). In addition, BMD can also be used to track changes in OP and evaluate the efficacy of OP drugs.

Numerous studies strongly suggest that, abdominal obesity, hypertension, dyslipidemia and dysglycemia are all considered as components of metabolic syndrome (MS) and are closely related to OP (Muka et al., 2015). Obesity may lead to an increase in bone density because it is associated with higher 17β-estradiol levels and higher mechanical loads, which may protect bones (Nelson and Bulun, 2001). Qiao et al. (2020) observed that adult obese patients had higher BMD in the lumbar spine and femoral neck than those of healthy weight. In general, obesity is negatively correlated with femoral neck OP, suggesting that obesity is a protective factor for OP (Qiao et al., 2020). Kim et al. (2010) found, however, that after adjusting for confounders, bone density was significantly lower in men over 40 and in postmenopausal women with MS. Moreover, the BMD decreased with the increase of MS components. Among MS indicators, waist circumference as a diagnostic criterion for abdominal obesity is the most critical factor leading to this negative correlation. Waist circumference was an important contributor in this association, suggesting that visceral fat may contribute to bone loss. The negative correlation between fat mass and bone density further supports this hypothesis, especially in men (Kim et al., 2010). Furthermore, pro-inflammatory molecules TNF- and IL-6 released from visceral fat play a key role in regulating bone resorption and participating in the pathogenesis of OP (Nanes, 2004; Roy et al., 2016).

Vitamin D is known to play a major role in the development and maintenance of bones and muscles in the body because of its ability to regulate the absorption of calcium and phosphorus. Low levels of vitamin D in the body are considered a potential risk factor for OP and bone fractures. Serum vitamin D deficiency prevents the intestinal tract from absorbing Ca^2+^ from diet, eventually leading to elevated levels of parathyroid hormone (PTH) secretion. Oversecretion of PTH induces osteoclast formation, inhibits osteogenesis, and maintains optimal blood calcium and phosphorus levels required for metabolic processes and neuromuscular function (Roy, 2013). Recent studies have found that the serum 25(OH)D of obese people is lower than that of normal weight people, which is negatively correlated with body weight, BMI, and fat mass (Fassio et al., 2018).

It is worth noting that recently, Li et al. proposed that obesity may lead to low serum 25(OH)D, high serum leptin and high bone density (Snijder et al., 2005; Konradsen et al., 2008; Felson, 2010). In this context, femoral neck and spine BMD were positively correlated with BMI and fat mass index. Recombinant human (Rh) leptin in the treatment of BMSCs significantly facilitated bone formation. In addition, leptin down-regulated CYP24A1 and up-regulated CYP27B1, CYP27A1, and VDR, all of which play key roles in vitamin D metabolism In summary, this study confirmed the relationship between obesity, vitamin D metabolism and osteoblastic development, and the direct effect of leptin on vitamin D metabolism and osteoblastic differentiation of BMSCs may protect bone under the effect of low serum 25(OH)D in obese people (Lim et al., 2019; Li J. et al., 2020).

### Fractures

Osteoporosis, sarcopenia, and obesity are commonly associated with aging. Obviously, fall-related injuries and fractures are the major causes of disability and death among the elderly, seriously affecting their quality of life and survival (Rapp et al., 2010; Sanchez-Riera et al., 2014).

Studies have shown that fat accumulation may contribute to the deterioration of muscle and bone, thereby promoting the development of sarcopenia and OP (Ilich et al., 2014). Additionally, more and more studies have confirmed that sarcopenia is not only closely related to low bone density, but also an important risk factor for fractures (Tarantino et al., 2015).

Obesity was previously deemed to be a protective factor for OP or brittle fractures because patients affected by obesity have more soft tissue to protect bone tissue. That is, the positive impact of mechanical load caused by body weight. But recent research suggests that obesity may increase the risk of certain fractures types (Cao and Picklo, 2015; Scott et al., 2016). Obesity may be a protective factor for hip fracture in adults and significantly reduce the risk of hip fracture (Tang et al., 2013). This view was driven in part by the positive correlation between BMD and BMI. Similar results were seen in obese patients with proximal femoral and vertebral fractures (De Laet et al., 2005).

The association between obesity and fracture in postmenopausal women may be site-dependent. Compared with normal/underweight women, obesity may prevent hip and pelvic fractures, but it increases the risk of proximal humerus fractures. Some non-spinal fractures, such as proximal humerus fractures, upper leg fractures, and ankle fractures, are at higher risk (Compston et al., 2011; Prieto-Alhambra et al., 2012).

In principle, obesity does not completely prevent fractures, and there are some specific site effects on fractures. In fact, obese people are more likely to fall and break bones than people of normal weight. Especially when BMI is over 30, obesity has limited protection against fractures and may even increase the risk of fractures (Kang et al., 2015).

### Osteoarthritis

Osteoarthritis (OA) is the most common degenerative joint disease that affects any joint in the elderly, especially the knee joint. OA is characterized by the progressive deterioration of articular cartilage and structural changes throughout synovial joints, such as synovial membrane, knee meniscus, adipose tissue, periarticular ligaments, and subchondral bone (Brandt, 2006; Loeser et al., 2012; Hunter and Bierma-Zeinstra, 2019). Clinical and animal studies have revealed that age-related OA is related to many factors including age, sex, trauma, and obesity. Among these factors, obesity is one of the most influential and modifiable risk factors (Bijlsma et al., 2011).

Actually, a growing body of evidence suggests a strong link between obesity and inflammation. Adipose tissue has been shown to regulate inflammatory immune responses in cartilage People and animals affected by obesity exhibit higher serum levels of TNF-α, IL-1 and IL-6, all from macrophages in adipose tissue (Park et al., 2005; Ding, 2011). In parallel, the levels of TNF-α, IL-1 and IL-6 in synovial fluid, synovial membrane, subchondral bone and cartilage in patients with OA were increased, confirming their important roles in the pathogenesis of OA TNF-α, IL-6, and IL-1 are the cytokines produced by adipose tissue to directly and negatively regulate cartilage. In addition, TNF-α, IL-1, and IL-6 can promote the formation of other factors, matrix metalloproteinases (MMPs) and prostaglandins, while restrain the synthesis of proteoglycans and type II collagen. Therefore, they play an important role in OA cartilage matrix degradation and bone resorption. Moreover, TNF-α, IL-1, and IL-6 may indirectly cause OA by regulating adiponectin and leptin secreted by fat cells (Koskinen et al., 2011; Wang and He, 2018; Tu et al., 2019).

Reyes et al. (2016) subsequently found that being overweight or obese increased the risk of OA in all three joint areas (knees, hips, and hands), especially the knees. Overweight, class I obesity and class II obesity increased the risk of knee OA by 2-, 3. 1-, and 4.7-fold, respectively (Reyes et al., 2016).

Adipokines represent a new class of compounds that are currently considered to be key molecules involved in the pathogenesis of rheumatic diseases (Felson and Chaisson, 1997; Scotece et al., 2011; Gremese et al., 2014; Feng et al., 2019). Resistin is an adipokines closely related to obesity, local low-level inflammation and MS (Rong et al., 2019). Alissa et al. (2020) recently found that serum resistin levels were higher in patients with primary knee arthritis than in healthy controls. In addition, elevated serum resistin levels were positively correlated with indicators of obesity, markers of inflammation, and WOMAC Index (an indicator of the severity of OA symptoms) (Alissa et al., 2020). Furthermore, Koskinen et al. (2011) believed that the combination of leptin and IL-1 could promote the production of MMP-1, MMP-3, and MMP-13 in human OA cartilage. The effect of leptin on MMP-1, MMP-3, and MMP-13 was mediated by transcription factor NF-κβ, and protein kinase C and MAP kinase pathways. Leptin concentration in synovial fluid was also positively correlated with MMP-1 and MMP-3 levels in patients with OA (Koskinen et al., 2011). The results showed that leptin had catabolic effect on OA joints by increasing the production of MMP in cartilage (Bao et al., 2010).

In addition, adiponectin has been reported to be involved in the pathophysiological process of OA. Kang et al. (2010) reported that the total amount of nitric oxide (NO) and the levels of MMP-1, MMP-3, and MMP-13 were increased in adiponectin stimulated OA chondrocytes compared with unstimulated cells. NO is one of the main mediators of pro-inflammatory cytokines acting on chondrocytes and also regulates different cartilage functions, including chondrocyte phenotypic loss, apoptosis, and extracellular matrix degradation (Otero et al., 2007). In this study, adiponectin increased the expression of MMPs and iNOS in human OA chondrocytes through AMPK and JNK pathways, leading to the degradation of OA cartilage matrix (Kang et al., 2010).

In summary, obesity not only increases the incidence of OA, especially in weight-bearing joints such as knee joints, but also is related to non-weight-bearing joints such as finger joints and wrist OA, suggesting that these metabolic mediators lead to an increase in the incidence of OA in obese patients. This may be because obesity increases the mechanical load of articular cartilage, leading to its degradation, and fatty tissue secretes metabolic factors (such as IL-1, TNF-A, adiponectin, and leptin), leading to an increased prevalence of OA in obese people (Oliveria et al., 1999; Grotle et al., 2008; Kalichman and Kobyliansky, 2009).

### Rheumatoid Arthritis

Rheumatoid arthritis (RA), the most common form of inflammatory arthritis, is a chronic systemic autoimmune disease characterized by aggressive symmetrical inflammation of multiple joints (Kobayashi et al., 2010; Miossec, 2013; Minamino et al., 2020). Epidemiological studies have shown that about 90% of RA patients develop bone erosion within 2 years of onset, resulting in joint deformity or even disability. Therefore, RA has brought a heavy burden and great pain to affected families, patients and even the whole society (Nam et al., 2017).

**FIGURE 1:**
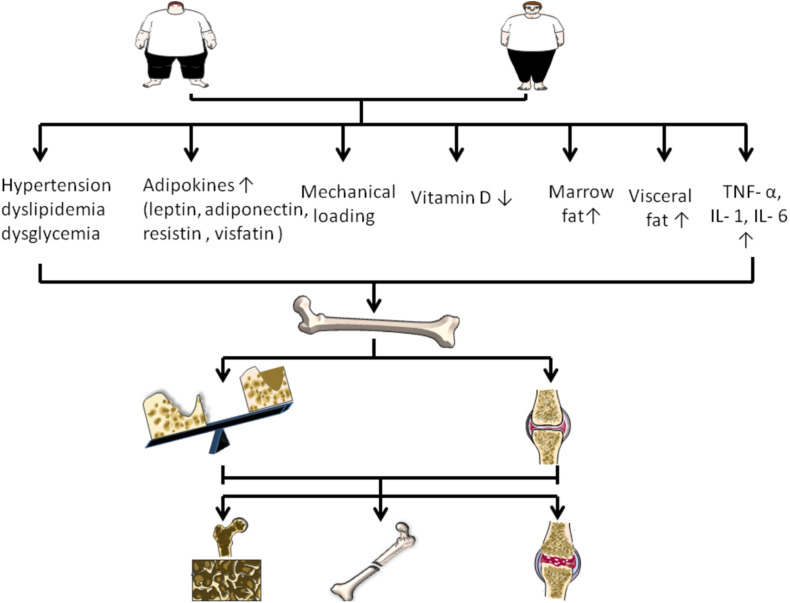
Changes of various factors caused by obesity on the regulation of bone disease. Obesity can increase mechanical load, visceral fat and bone marrow fat. In addition, obesity is associated with increased adipokines, increased TNF – or, IL-1, IL- 6, decreased vitamin D, and accompanied by hypertension, dyslipidemia, and dysglycemia. They regulate bone disease by affecting bone formation, bone resorption, and cartilage.

Overweight/obesity is associated with higher rates of chronic autoimmune diseases and inflammatory diseases, including type 2 diabetes and RA (Zhang et al., 2014). There is evidence that an increase in BMI is associated with an increased risk of RA (Feng et al., 2019). As mentioned above, adipokines such as adiponectin and visfatin have also been reported to play a key role in the pathophysiology of autoimmune diseases (Coelho et al., 2013). It has now been well established that patients with RA show higher plasma adiponectin, leptin, and visfatin levels compared with healthy controls (Otero et al., 2006). Visfatin is a proinflammatory mediator that induces the production of TNF-α, IL-1, IL-6, IL-8, and MMPs, which are typical manifestations of RA joint inflammation (Brentano et al., 2007). Similarly, adiponectin stimulated fibroblast-like synoviocytes (FLS) in patients with RA to produce IL-6, IL-8, and PGE2 (Choi et al., 2009; Lee and Bae, 2018). In addition, adiponectin increased the production of VEGF and MMPs in RA FLS, which may induce inflammation and joint destruction (Lee et al., 2014; Choi et al., 2020; Figure 1).

Previous studies have shown that the frequency of circulating T follicular helper cells (Tfh) is significantly increased in RA patients, which is positively correlated with disease activity and anti-CCP autoantibody levels (Liu et al., 2012, 2015). RA FLSs stimulated by AD (adiponectin) promoted the production of Tfh cells. In addition, intra-articular injection of AD aggravated synovitis and increased the frequency of Tfh cells in CIA mice treated with AD (Nurieva et al., 2008; Liu et al., 2020).

Obesity is not only prevalent in RA patients, but also associated with disease activity. Obesity reduces the chance of RA remission and negatively affects disease activity and outcomes reported by patients during treatment (Liu et al., 2017). Lee and Bae (2018) observed that the levels of circulating adiponectin and visfatin in RA patients were significantly higher than those in the control group. The levels of visfatin in 28 joints were positively correlated with disease activity score and CRP level (Lee and Bae, 2018).

## Obesity Type and Bone

On the one hand, obesity is divided into peripheral obesity and abdominal obesity according to the distribution of fat in the body. Abdominal fat is made up of abdominal wall fat (SAT) and abdominal fat (VAT), also known as central obesity, visceral obesity. Previous studies have shown that adipokins are associated with bone metabolism, and that central obesity can lead to osteopenia or OP because bone density decreases with an increase in waist-to-hip ratio, an index of central obesity (Mitsuyo et al., 2007). In one study, whole body bone mineral content was positively correlated with HOMA-IR and negatively correlated with the percentage of trunk fat, which is a good representative of visceral fat, suggesting that abdominal obesity may have an adverse effect on systemic bone parameters (Krishnan et al., 2018).

Local fat is increasingly recognized as a determinant of bone density, and this association may be mediated by adipocytokines (Vicente et al., 2009). Russell et al. (2010) proposed that VAT was a negative predictor of spine BMD, apparent BMD, systemic BMD and bone mineral content for obese adolescent girls aged 12–18 years (Katzmarzyk et al., 2012). Importantly, VAT/SAT, adipokines, cytokines, E-selectin, and adiponectin were negative predictors of bone density, while leptin was positive. Consequently, VAT is an independent negative determining factor of bone density in obesity (Jurimae et al., 2008; Agbaht et al., 2009; Russell et al., 2010; Bredella et al., 2011a).

**FIGURE 2:**
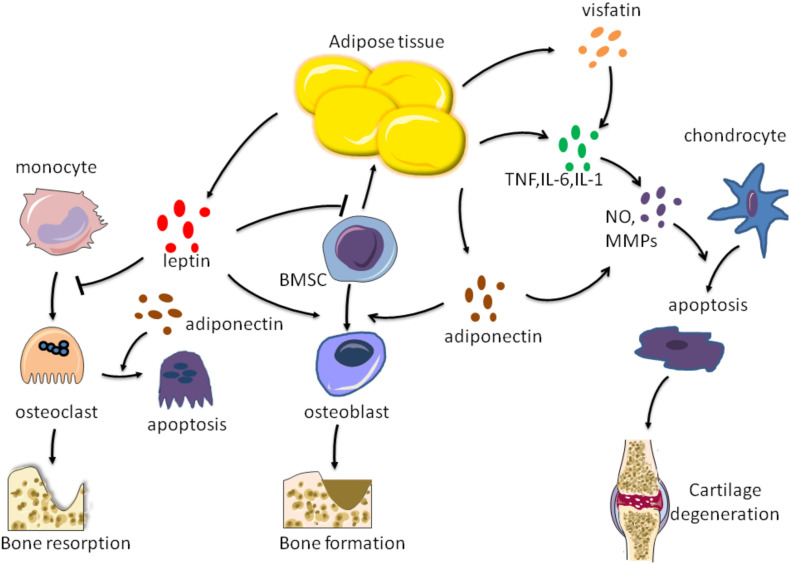
Effects of factors secreted by adipose tissue on bone metabolism. Adipose tissue can secrete leptin, adiponectin, visfatin, TNF-*a*, IL-6, and IL-1 These factors act on chondrocytes, osteoblasts, osteoclasts, respectively, to regulate bone formation and resorption, as well as cartilage degradation.

On the other hand, according to the different obesity phenotypes, it can be divided into normal metabolic healthy BMI, metabolic healthy obesity and metabolic abnormal obesity (Karelis et al., 2004; Dobson et al., 2016). Marques Loureiro et al. (2019) observed significantly increased deficiencies of calcium, phosphorus, vitamin D, and PTH in the metabolically unhealthy obese (MUHO) group compared to the metabolically healthy obese (MHO) group. In summary, the MUHO phenotype presents a higher risk of bone metabolism-related changes, which may contribute to the development of metabolic bone disease (Marques Loureiro et al., 2019; Figure 2).

## Different Populations With Obesity and Bone

### Children and Adolescents

While childhood obesity has always been a major health problem, its prevalence has been on the rise. In addition, childhood obesity may be associated with multiple complications, such as hyperinsulinemia, hypertension, MS, and non-alcoholic fatty liver disease (NAFLD; Oh et al., 2016). Childhood obesity may affect the growth patterns of children and adolescents, according to several studies Children influenced by obesity may develop accelerated skeletal maturity and advanced bone age beyond their actual age (Johnson et al., 2012; Marcovecchio and Chiarelli, 2013). A study of 232 children (aged 6–15 years) found that the prevalence of advanced bone age increased significantly with increased body weight, height, BMI, and waist circumference percentiles (Oh et al., 2020).

Instead, a study of young people with an average age of 10–17 confirmed that obese children and adolescents had higher bone mass and density than their normal-weight peers (Chaplais et al., 2018).

Notably, Zhao et al. (2020) recently found that obesity had no benefit for BMD in Chinese children aged 0-5 years. t BMD was positively correlated with age, height/length, and inversely with BMI. The BMD gradually increased in the range within 21.2kg, but began to gain slowly and even decreased when the body weight exceeded 21.2kg (Zhao et al., 2020).

As noted above, although there have been several studies on the effects of fat mass on skeletal health in normal weight and obese adolescents, the results remain controversial.

### Postmenopausal Women

Osteoporosis is considered a major public health problem for postmenopausal women. Low estrogen levels lead to rapid bone loss in women five to seven years after menopause (Kanis et al., 2008). Actually, some evidences, indicated that age and BMI were important factors influencing BMD. The BMD of obese postmenopausal women was higher than that of normal size women, and the reduction of BMD of obese women can be delayed by weight bearing (Méndez et al., 2013).

At the same time, Cherif et al. also observed that the left femur, right femur, total hip joint, and overall bone density were higher in obese women (Cherif et al., 2018).

In addition, adipokines secreted by fat are considered as potential pathophysiological factors of OP. Several studies have shown that leptin has significant effects on bone growth and bone metabolism through central and peripheral pathways, and may be involved in the occurrence of various bone diseases (Chen and Yang, 2015). Studies have shown a positive correlation between leptin levels and BMI. And higher BMI is associated with higher bone density. However, obesity had no effect on adiponectin and resistin secretion in postmenopausal women with OP, so leptin was the only one of the adipokines studied to be considered as a protective factor for bone tissue in postmenopausal women (Pasco et al., 2001; Glogowska-Szelag et al., 2019).

Thus, the above results indicate that, adiposity may be beneficial to bone density in postmenopausal women. The protective effect of high body weight and BMI may be due to hormonal influences in the body. Postmenopausal women affected by obesity have more adipose tissue and more estrogen conversion, resulting in higher estrogen levels in their bodies.

### Elderly Patients With Obesity

Obesity, sarcopenia, and OP are common chronic diseases in the elderly. Sarcopenia is a newly discovered age-related disease related to lipid metabolism and insulin resistance. The main diagnostic criteria for sarcopenia are reduced skeletal muscle mass, muscle strength, and function. Older people continue to lose muscle mass as they age, while body fat, especially visceral fat, tends to rise, known as "sarcopenic obesity" (SO; Stenholm et al., 2008; Arango-Lopera et al., 2013). A recent study found that women with SO were more easy to show elevated blood glucose, while men with SO were more likely to present with OP and dyslipidemia (Du et al., 2019). On the other hand, muscles secrete a set of cytokines called myokines, thereby regulating bone metabolism. Myostatin, as a key myokine, has been reported for its effect on bone. Myostatin can inhibit osteogenic differentiation of BMSCs, as well as osteoblast differentiation and mineralization (Hamrick et al., 2007; Chen et al., 2017). Likewise, myostatin may inhibit osteogenesis by activating the RANKL signaling pathway, thus showing an adverse impact on bone mass (Saad, 2020). In addition, myostatin itself is an important autocrine/paracrine factor that inhibits skeletal muscle growth (Rodriguez et al., 2014; Cui et al., 2020). Thus, inhibition or blocking of the myostatin signaling pathway may provide potential therapeutic targets for a number of diseases, particularly in sarcopenia and OP.

Several studies have reported the links between BMD and body fat and lean mass. When body weight was stratified into lean body mass and fat mass, the increase in BMD was more pronounced for lean body mass, whereas fat mass was only beneficial for men and premenopausal women. Santos et al. also observed a more direct relationship between lean body mass and bone density (total bone density, femur, and spine), while sarcopenia was associated with OP. Obesity was more likely to be a protective factor for OP in old subjects aged 80 and over (Santos et al., 2018). At the same time, Barrera et al. demonstrated the beneficial effects of high BMI on femoral neck bone density in older adults. Men and women with a BMI of more than 30 kg/m^2^ had about a 33% risk of bone mass loss compared to those with a normal BMI (Barrera et al., 2004). In particular, obese people were reported to have higher bone density, but they also showed damaged bone microstructures and different fall patterns (Compston, 2013; Ilich et al., 2016).

## High-Fat Diet Induced Obesity and Bone

Conclusions about the relationship between obesity and bone in humans rely on statistical correlations or models, rather than controlled trials. Therefore, the establishment of obesity mouse model is helpful to study the effect of high-fat diet (HFD)-induced obesity on bone metabolism. Studies have shown that obese animals burn the same amount of energy, no matter how much fat is in their diet (Brown et al., 2003; Tortoriello et al., 2004; Relling et al., 2006). The mice provided a model for studying the relationship between body size, obesity and skeletal characteristics. High fat intake in rodents leads to obesity, and several studies have shown a strong link between bone size, strength and body size. However, mice are not always reliable indicators of human pathophysiology. Human can enjoy more colorful life style, more abundant food and more complicated living environment. Moreover, patients with obesity often have multiple complications, not just weight gain. These factors make the relationship between obesity and bone more complex in humans than in mice.

### Cancellous Bone

The effects of a high-fat diet on cancellous bone in rodents have been shown to be harmful. Previous studies have reported that after 4, 8, and 12 weeks of HFD treatment, the trabecular density of 6-week-old male C57BL/6J mice decreased with the increase of adipocytes and trabecular degeneration. In addition, in obese mice, serum leptin levels were associated with bone trabeculae, but not cortical bone density, while adiponectin and total cholesterol levels were not associated with bone mass (Fujita et al., 2012). Scheller et al. (2016) also noted that bone trabecular volume fraction, bone mineral content, and quantity decreased after 12, 16, or 20 weeks of high-fat feeding compared with normal rat chow (ND) controls, and only partial recovery after weight loss.

In addition, Inzana et al. (2013) found that femoral cancellous metaphyseal bones were more susceptible to adverse effects of high-fat diet before bone maturation, and had poor recovery ability after dietary correction (low-fat diet, LFD).

A recent study conducted by Tian et al. showed that the bone mass of femoral trabecular bone in C57BL/6J mice increased significantly after 8 weeks of HFD, but decreased significantly at 16 and 24 weeks (Tian et al., 2017). In other words, after short-term feeding, HFD may show a positive effect on bone mass, however, after long-term feeding, bone mass was significantly decreased in HFD mice.

### Cortical Bone

However, the effects of diet induced obesity on cortical bone in rodents are less clear, with positive, negative, and neutral results reported. The femoral cortical thickness and cross-sectional area of 4-week old male mice were increased after feeding HFD-DAG (Diacylglycerol). HFD-DAG had obvious promoting effect on bone and bone metabolism (Choi et al., 2015). In addition, Silva et al. recently suggested that a high-fat diet had beneficial effects on most femoral size and skeletal mechanical properties, as well as radius size and stiffness (Silva et al., 2019). However, Ionova-Martin et al. found that femur strength, hardness, and toughness were significantly lower in both young and adult mice fed HFD than in the control group (Ionova-Martin et al., 2011).

In contrast, Cao et al. concluded that feeding mice HFD for 14 weeks reduced proximal tibial cancellous bone mass in young mice, but had no effect on cortical bone mass (Cao et al., 2009). Halade et al. (2011) also revealed that mice fed corn oil (CO) for 6 months showed a 66% reduction in distal femoral trabecular volume fraction, with no significant effect on cortical bone. To sum up, in the above studies, the effect of HFD on cortical bone was not as significant as that on cancellous bone.

It is generally believed that age-related OP has three main processes. The first and most important process is reduction of trabecular bone, the second is continuous bone resorption on the cortical surface, and the third is cortical bone loss (Chen et al., 2009). Similarly, the above studies indicate that the most significant change in obesity-related bone loss is the reduction of femoral trabecular bone Combined, these results suggest that HFD could regulate the changes of trabecular and cortical bone in different ways. This may be due to the fact that cancellous bone generally responds more strongly to diet or drug therapy, physiological conditions, or aging than cortical bone, because cancellous bone is more active in remodeling because of its larger surface to volume ratio than cortical bone (Morgan et al., 2008). On the other hand, bearing capacity and mechanical stress are important factors in determining cortical bone mass, while trabecular bone density is affected by sex maturation related hormones (Mora et al., 1994).

### Bone Formation/Resorption

In addition to affecting bone structure, HFD can also have significant effects on cell function. Bone mass reflects the balance between bone formation and bone resorption and is involved in the coordination and regulation of the number and activity of osteoblasts and osteoclasts at the cellular level. The RANKL/RANK/OPG signaling pathway plays a major role in this regulation. A previous study showed that the expression of RANKL, the ratio of RANKL to OPG, and the level of serum TRAP in osteoblasts from HFD mice were increased, suggesting that HFD can promote osteoclast activity and bone resorption (Cao et al., 2009). Notably, Halade et al. reported that in mice fed HFD, the accumulation of bone marrow adipocytes resulted in significantly higher levels of pro-inflammatory factors, leading to increased bone resorption. In addition, increased expression of osteoclast-specific cathepsin K and RANKL and decreased osteoblast-specific RUNX2/Cbfa1 in CO-feeding mice also supported bone resorption (Halade et al., 2011).

Furthermore, Shu et al. (2015) revealed that the lower trabecular volume, but increased osteoclast numbers could be found in the femoral metaphyseal sections of HFD-fed mice after 3, 6, and 12 weeks. The elevated osteoclast precursor frequency, increased osteoclast formation, and bone resorption activity, along with increased osteoclastogenic regulators such as RANKL, TNF, and PPARγ were seen in bone marrow cells from HFD-fed mice. But, osteoblast function was also increased after 12 weeks of HFD (Shu et al., 2015). A possible explanation is that mechanical load of body weight stimulates bone formation, reduces apoptosis, and enhances proliferation and differentiation of osteoblasts and osteocytes. Therefore, it was not surprising that bone formation rates and osteoblast numbers increased in this study, since HFD mice were significantly heavier than the control group (Bonewald and Johnson, 2008). In conclusion, it is reasonable to believe that the bone loss caused by HFD is mainly related to the promotion of osteoclast differentiation and activity by changing the bone marrow microenvironment.

Based on these findings, the effect of HFD is bipolar and may be the result of a combination of body weight, fat mass, bone formation/absorption, pro-inflammatory mediators and bone marrow microenvironment. Obesity initially has a beneficial effect on bones, possibly due to anabolic effects that increase mechanical load. However, due to the development of metabolic complications including systemic inflammation, the second stage is followed by a reduction in bone formation (Lecka-Czernik et al., 2015). As mentioned above, these results may support the idea that as obesity rises, the benefits for bone health are diminishing.

## Conclusion

In conclusion, obesity or overweight is strictly related to bone metabolism, although the correlation has not yet been fully unified. Adipose tissue interacts with bone by secreting various cytokines, so as to regulate bone health. Meanwhile BMAT also exerts a crucial impact on bone density and bone microstructure. In addition, human obesity is a complex problem that involves not only excessive fat intake but also other nutrient consumption imbalances such as vitamin D, calcium and phosphorus, which are known to affect bone metabolism, further making it difficult to determine the impact of obesity on human bone health. Moreover, while BMI is closely related to the gold standard of body fat, it does not distinguish between lean and fat mass, nor does it provide an indication of the distribution of body fat. The loss of muscle mass in the elderly means that BMI is also less accurate at predicting body fat in this group. Therefore, determining whether obesity causes changes in bone mass based on BMI is less accurate. Central obesity measures, including waist circumference, waist-to-height ratio and waist-to-hip ratio, are better predictors of visceral obesity, bone-related disease and mortality than BMI. Simply put, all of these findings indicate that skeletal response to obesity has either a positive or negative effect on bone, suggesting that the influence of obesity on bone metabolism is intricate and depend on diverse factors, such as mechanical load by the weight, obesity type, the location of adipose tissue, gender, age, and bone sites, along with secreted cytokines, these factors may play a major function for bone health. The effects of obesity on bone metabolism and bone microstructure involve these multiple factors, which may exert different regulatory mechanisms and ultimately affect the skeletal health. The investigation of the relationship between obesity and bone is conducive to finding new targets for the treatment of bone-related diseases, including OP, fractures, RA, and OA.

## Author Contributions

JH wrote the manuscript. CH, WH, and MY revised the manuscript. CL and XL were responsible for the guidance and supervision. All authors contributed to the article and approved the submitted version.

## Conflict of Interest

The authors declare that the research was conducted in the absence of any commercial or financial relationships that could be construed as a potential conflict of interest.
